# Underestimation of Human Cutaneous Leishmaniasis Caused by *Leishmania infantum* in an Endemic Area of the Mediterranean Basin (Balearic Islands)

**DOI:** 10.3390/microorganisms11010126

**Published:** 2023-01-04

**Authors:** Maria Magdalena Alcover, Vicenç Rocamora, Alexis Ribas, Roser Fisa, Cristina Riera

**Affiliations:** 1 Parasitology Section, Department of Biology, Healthcare and Environment, Faculty of Pharmacy and Food Science, University of Barcelona, 08028 Barcelona, Spain; 2 Department of Dermatology, Manacor Hospital, 07500 Manacor, Spain; 3 Institut de Recerca de la Biodiversitat (IRBio), Universitat de Barcelona, 08028 Barcelona, Spain

**Keywords:** *Leishmania*, cutaneous leishmaniasis, Majorca, subnotification of cases

## Abstract

Leishmaniasis is an infectious zoonotic disease caused by protozoan parasites of the genus *Leishmania*. In the Mediterranean basin, leishmaniasis is caused by *Leishmania infantum* and transmitted by bites of sandflies of the genus *Phlebotomus*, with the dog as the main reservoir host. The most common form is cutaneous leishmaniasis (CL), although visceral cases also occur. The aim of this study was to assess the underestimation of CL in an endemic Mediterranean region. Thus, a retrospective study was performed on all CL cases diagnosed and treated in the Dermatology Service of Manacor Hospital (Majorca, Balearic Islands), and the data obtained were compared with those of local government epidemiological bulletins for the same period. The different clinical presentations were compiled, and data related to sex, age, and lesion type and number were analyzed. The results reveal a clear sub-notification, which indicates that the real incidence of human CL in this area is unknown.

## 1. Introduction

A widespread yet neglected parasitic disease, leishmaniasis constitutes a major public health problem in many regions with tropical or subtropical climates, including the Mediterranean basin. The causal agents are different *Leishmania* species, which are transmitted through the bite of nematoceran dipterans of the subfamily Phlebotominae: the genus *Phlebotomus* in the Old World and *Lutzomyia* in the New World. About 70 species of animals, including humans, are affected [1]. In the Mediterranean area, leishmaniasis is a zoonosis, while in other geographical areas, it is zoonotic or anthroponotic [1]. The different clinical forms of the disease depend on the *Leishmania* species and its tropism, the interaction between host and parasite, and the host’s immune response.

According to the World Health Organization (WHO), 95% of cutaneous leishmaniasis (CL) cases in 2018 were located in America, the Mediterranean basin, the Middle East, and Central Asia. In the same year, more than 85% of new CL cases occurred in Afghanistan, Algiers, Bolivia, Brazil, Colombia, the Islamic Republic of Iran, Iraq, Pakistan, the Syrian Arab Republic, and Tunisia. Each year there are between 50,000 and 90,000 new cases of visceral leishmaniasis (VL), which without treatment, is fatal in 95% of patients, and between 600,000 and 1 million new cases of CL [1]. Leishmaniasis, in cutaneous and visceral forms, is hypoendemic in Mediterranean countries [2]. In Spain, both are caused by *Leishmania infantum*, which is transmitted by the sandflies *Phlebotomus ariasi* and *P. perniciosus*, with the dog as the main reservoir [1,2].

The declared prevalence of leishmaniasis in Spain is 0.3 cases per 100,000 inhabitants, and the autonomous communities most affected are Valencia, Madrid, Catalonia, and the Balearic Islands. Although leishmaniasis has been a notifiable disease since 2015, its changeable notification status in prior years has led to controversies in the available epidemiological data. From 2014 to 2017, 1359 autochthonous cases of VL and CL were reported nationwide, with the highest incidence of CL found in the Balearic Islands (2.13 cases/100,000 inhabitants) and the Valencian Community (1.16 cases/100,000 inhabitants) [3]. 

The Spanish autonomous community of the Balearic Islands is an archipelago located in the western Mediterranean comprising four main islands (Majorca, Minorca, Ibiza, and Formentera) as well as uninhabited islets. The largest is Majorca, which covers 3640 km^2^ and has a coastline of 623 km, with a population of 1173,008 in 2021 [4]. The most recent local government epidemiological surveillance reports, corresponding to 2017 and 2018, indicate that Majorca was the Balearic Island most affected by leishmaniasis, having 78.5% of declared cases of CL in 2017 and 77% in 2018 [5,6,7,8,9]. In a study carried out on blood donors from the Balearic Islands, *L. infantum* DNA was detected in peripheral blood mononuclear cells in 18 of 304 patients (5.9%) [10]. 

Clinical signs and symptoms of CL may differ depending on the causative agent, and they generally follow a similar pattern within a geographic area [11,12]. In the Mediterranean region, where the main causative species is *L. infantum*, CL occurs most frequently in the acute form, which involves an evolution of less than 1 year in zoonotic infections (and less than 2 years in anthroponotic infections) [13].

CL is manifested by the appearance of one or more non-painful ulcerative lesions in uncovered areas of the body, especially the face, neck, arms, and legs. A nodule appears at the point of inoculation, which may develop into a painless ulcer. The typical lesion is an erythematous papule that evolves over weeks into a crusted, ulcerated, nodular form or plaque known as the Oriental sore. Diagnosis based on histopathology reveals a chronic non-necrotizing granulomatous infiltrate formed by plasma cells and lymphocytes, which may require further analysis to rule out granulomatous conditions such as sarcoidosis, tuberculosis, leprosy, syphilis, and deep mycosis [14].

Multiple lesions can sometimes be seen. The occurrence of hypoesthetic, psoriasiform, eczematous, varicelliform, verrucous, or even keloidal variants entails differential diagnosis to discard other dermatological conditions. Most acute CL lesions heal in the months following the sandfly bite, although a small percentage evolve into chronic or disseminated CL [15].

CL can also develop from mucosal inoculation, produced by a bite on exposed mucosa such as the nose, lip, tongue, or mouth. It does not have a single form of presentation and may resemble other conditions, such as a boil on the nasal mucosa, angioedema of the lip, or granulomatous cheilitis. This type of infection should be differentiated from CL that secondarily affects the mucosa or mucocutaneous forms because the course and prognosis are different [15]. 

Chronic CL usually affects adults and the elderly due to their particular immunological conditions, commonly manifesting as a chronic skin ulcer without the presence of parasites, although there is also a form without ulceration [15].

Susceptibility to the disease depends on the conditions of the exposed individuals, the endemic area, and the *Leishmania* species involved. Due to the important role played by cellular immunity in protection against *Leishmania*, the disease is developed most frequently by individuals with immunodeficiencies (due to immunosuppressive treatments, hematological malignancies, autoimmune diseases, and HIV seropositivity) and children [16].

To make a correct diagnosis of CL, three criteria should be considered in the following order: epidemiological history, clinical picture suggestive of leishmaniasis, and parasitological diagnosis. The parasite must be confirmed by visualization (positive parasitology by staining and/or culture of the lesion) or detection of DNA (polymerase chain reaction (PCR) assay) in the lesion exudate or biopsy. Molecular diagnosis is generally useful for confirming CL, particularly when other techniques give negative results. Moreover, molecular techniques such as PCR-restriction fragment length polymorphism (RFLP) can differentiate between *Leishmania* species. The serological tests are not usually suitable for CL because the antibody levels are undetectable or very low. Only for mucocutaneous leishmaniasis can positive serology (immunofluorescence antibody test, enzyme-linked immunoassay) be accepted as a diagnosis. It should also be considered that the accuracy of many diagnostic methods is highly variable [17,18].

The objectives of this study were a) to determine the real extent and possible under-notification of CL in an area of the Balearic Islands by comparing the cases diagnosed in the Dermatology Service of the Manacor Hospital with those notified in the Epidemiology Service of the Balearic Islands and b) to describe the pattern of presentation of leishmaniasis in patients from the Balearic Islands.

## 2. Materials and Methods

### 2.1. Study Area Context

Opened in 1997, the Manacor Regional Hospital (Majorca) forms part of the Health Service of the Balearic Islands, which depends on the Department of Health of the Government of the Balearic Islands. It has a staff of more than 1000 and more than 200 hospital beds. Situated in the county of Llevant [19], the hospital provides care for a population of about 150,000, which in the summer is increased by a floating population of more than 20,000, and has an area of influence of 248,548 ha [20,21]. 

### 2.2. Data Collection and Clinical Sampling

A retrospective study was carried out of all CL cases diagnosed and treated in the Dermatology Service of Manacor Hospital (Majorca, Balearic Islands) between 2013 and 2017. Patient medical records, as well as the records of the Microbiology and Pathology Departments, were reviewed to identify the patients and compile the test results. The selection criteria for including cases in this study were a positive parasitological test and/or positive PCR. For direct parasite observation, amastigotes were visualized on a Giemsa-stained exudate/biopsy smear and/or in histological sections of the biopsy by means of hematoxylin and eosin staining, which was performed in the Department of Microbiology and Pathological Anatomy of Manacor Hospital. Parasite DNA was detected by real-time PCR (RT-PCR) on samples freshly collected and/or on Whatman filter paper in the Section of Parasitology at the Faculty of Pharmacy of the University of Barcelona [22,23]. Positive samples suspected to involve species other than *L. infantum* due to patient origins or recent travels were tested by a PCR-RFLP method [24,25]. Although no standardized diagnostic protocol was followed, confirmation tests for CL in different types of samples were taken into consideration (observation and/or isolation of parasite and/or PCR-positive test). Cases that did not meet these requirements were excluded. 

Clinical lesions were classified as single or multiple (co-existence of two or more), and lesion type, size, and location in the body were recorded. 

To determine whether CL is underreported in the study region, the number of notified CL cases in the Balearic Islands was obtained from epidemiological bulletins of the Autonomous Government of the Balearic Islands [26] and compared with the number of cases identified in this retrospective study over the same period. The estimated incidence of CL in the county of Llevant was calculated based on the number of cases diagnosed in the Manacor Hospital (the only public hospital in the region) and the population census in the same region.

### 2.3. Ethics

All patients signed an informed consent form before the diagnostic tests were performed. The standard “Dermatology Informed Consent” form of the Manacor Hospital was used for “surgical intervention or other diagnostic/therapeutic procedures or taking photographic images to monitor the pathology”. In addition to stating that they are aware of the reasons, convenience, advantages, and risks of the proposed intervention or procedure, the patient authorized the taking of photographs to monitor their case or for scientific purposes. Informed consent revocation forms were also available. 

## 3. Results

According to the notification system of the local health authorities, in the period 2013–2017, there were 141 cases of human leishmaniasis in the Balearic Islands, 109 of them CL. In contrast, the retrospective analysis of CL diagnosed in Manacor Hospital of Majorca revealed as many as 126 cases only in the county of Llevant for the same period (Table 1). 

A case-by-case review of the data from the Manacor Hospital revealed that only 27.8% of the 126 cases were officially notified, which implies that more than 70% of cases remained unreported.

The clinical profile of 41 of the 126 patients led to the suspicion of diseases other than CL, most commonly basal cell carcinoma, squamous cell carcinoma, mycobacterial infection, and nevus (three cases each), followed by furuncle, lymphoma, and ringworm (two cases each) (Table 2).

Regarding sex, a slight predominance of women was found (about 52%). Differences were also observed in the distribution of sex by age group, with more males among patients under 18 years of age and more females among those over 65. Analyzing the frequency of infection according to age, the group with the most cases was 18–65 years (50.8%), followed by 65 years or older (26.2%), and under 18 years (23%) In the latter group, 16 patients (55.1%) were between 0 and 5 years old, 10 (34.5%) between 6 and 11, and 3 (10.4%) between the ages of 12 and 16 (see Table 3 for details).

The associations of lesion type with sex and age in a total of 161 lesions are shown in Table 4. Single lesions predominated (102/161; 81%) [95% confidence interval (CI) 73.2–86.9] in both sexes, with a higher frequency in women, and in all the age groups. In patients under 18 years of age, 75.9% (95% CI: 57.6–88) had a single lesion, and 24.1% (95% CI: 11.9–42.3) had multiple lesions. In the 18–65 age group, 76.5% (95% CI: 64.7–85.3) presented a single lesion, 23.5% (95% CI: 12.1–31, 8) had multiple lesions, and 3.2% (95% CI: 0.24–11.60) a single mucosal lesion. Single lesions were far more frequent in the group over 65 years of age: 94% (95% CI: 79.4–99.3) compared to only 6% with multiple lesions (95% CI: 0.6–20.6). In the examined series, the location of the first and second lesions coincided in 14 (64%) of the 22 patients with multiple lesions (Table 4).

The head and neck were the most frequent location of single and first lesions (when multiple), followed by the upper extremities, whereas few were found in the lower extremities. The head and neck were the most affected area in both sexes and all age groups. In this location, single papular lesions were the most frequent, and there were no major differences regarding the number of papules and plaques. In children, almost all lesions were in the head and neck, with none in the upper extremities. In elderly patients, no lesions were observed in the trunk. The type and number of lesions in each area according to patient sex and age are summarized in Table 5.

Regarding the size of the lesion related to sex and age, those from 6 to 10 mm were the most frequent in both sexes and all age groups (Table 6), representing more than half of single or first lesions. Lesions of 11 to 15 mm and 1 to 5 mm were less frequent, and there were few cases of lesions larger than 16 mm.

**Figure 1:**
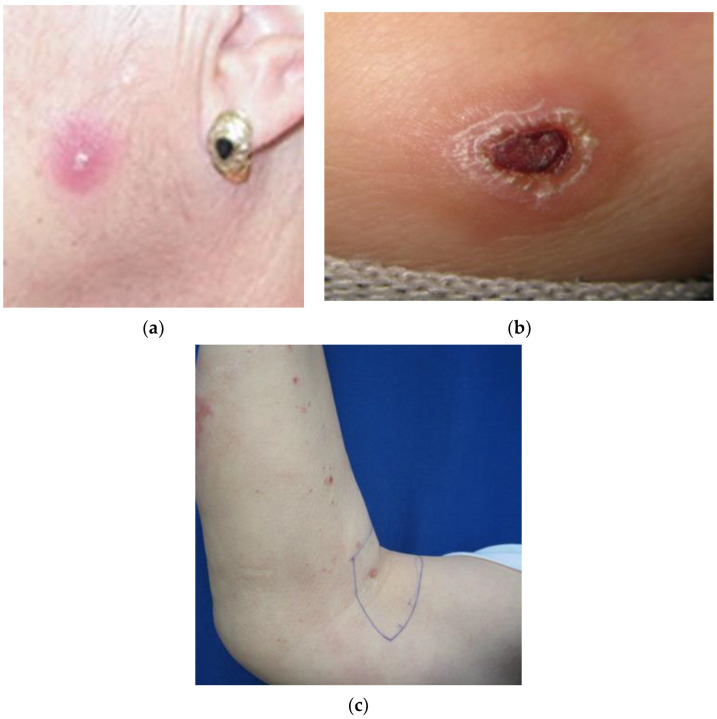
Cutaneous leishmaniasis lesion type: (**a**) single papule; (**b**) plaque with crust; (**c**) multiple lesion papule.

The mean size of single or first lesions was 10 mm, and second, third, and fourth lesions were 8, 8, and 11 mm, respectively. By sex, considering all the lesions, the mean size was 7.6 mm in women and 10.2 mm in men (Table 6, Figure 1).

## 4. Discussion

Leishmaniasis is a notifiable disease in the Balearic Islands. As the official epidemiological bulletins provide data without specifying individual islands, a direct comparison with the number of CL cases found in the Manacor Hospital records could not be made. Instead, the CL incidence in the study area was estimated from the bulletins and compared with the retrospective data obtained.

To calculate the annual incidence of CL in the county of Llevant (cases per 100,000 inhabitants), the 126 cases in the study were classified according to the year of diagnosis. In the Manacor Hospital, the diagnosis and notification of CL increased during 2013–2017, which could be attributed to a greater awareness of the disease due to the findings of a final degree project carried out in this health center [27]. 

Underreporting of CL can be related to several factors: it is not a very serious disease, distant mucosal involvement or derived VL is not usually visible, and the treatment is effective. Although CL is more frequent than the visceral form, more cases of VL are reported because of its higher morbidity. Another factor that could influence underreporting, although it is only a hypothesis based on experience, is the high care burden suffered by the public health system. 

Leishmaniasis, especially CL, is described as occurring more frequently in children. A higher incidence in childhood could be explained by the immaturity of the immune system, longer exposure to the vector [28], and greater penetrability of the skin, which would facilitate parasite inoculation by the vector [29]. However, in our study population, CL does not appear more prevalent in children than adults, as 77% of the patients were older than 18 years. This could be because of the spontaneous healing of lesions in minors, avoiding the need for hospital treatment. Another possible cause is that not all the necessary diagnostic tests were performed [30]. Other studies in Spain have reported variable results with respect to the incidence in children. A study in Granada in 1989 found that 56% of patients were under 5 years of age [31], but in Madrid in 1990, children were a minority, with 38% of patients under 12 years [32]. Similarly, in Toledo, 30% were under 14 years of age [28], as were 8% in Fuenlabrada (Community of Madrid) [29]. 

The slight majority of female patients in the present study is in agreement with a report from Madrid, where 17 of 31 CL patients between 1981 and 1989 were women (54.83%) [32]. On the other hand, a clear majority was observed in a tertiary hospital in Toledo, with 30 of 43 patients with CL between 1990 and 1997 (70%) being women [28]. In Fuenlabrada, 71 of 116 patients (48%) were female [29]. 

The predominance of single rather than multiple lesions found in this research matches published results for other endemic areas of Spain [28,31,32,33]. In contrast, in an unusual epidemic outbreak in Fuenlabrada between 2010 and 2012, multiple lesions predominated [29]. The appearance of single or multiple lesions depends on different factors, such as the species or strain of *Leishmania*, the vector, and the genetic predisposition of the individual [34].

Single lesions occurred in the majority of patients in all age groups, generally with an equal proportion of papules and plaques, except in individuals aged 18 to 65 years, where there was a slight predominance of plaques. A predominance of single lesions in patients under 18 years of age has also been observed in other studies [28,31,35].

Regarding the mean lesion size, our data coincide with other reports in Spain [28,31,32]. Again, the cases in the Fuenlabrada outbreak follow a different trend, with 33% of the lesions being smaller than 5 mm [29]. 

In patients with multiple lesions, we found that 36% (8/22) were affected in different body areas, compared to 16% in the Fuenlabrada outbreak [29]. Cases of multiple lesions are most likely caused by several bites from a single sandfly that fails to extract blood in the first attempt and deposits promastigotes with each bite [36]. Other explanations for multiple lesions are simultaneous bites from several sandflies [37] or lymphatic spread [34].

## 5. Conclusions

Although a notifiable disease, CL is not usually reported. The number of CL cases diagnosed in a single hospital on the island of Majorca between 2013 and 2017 was higher than all the cases reported in the Balearic Islands for the same period. The underreporting of CL could be related to several factors: it does not have serious outcomes; the clinical presentations are highly variable; distant mucosal involvement or derived VL is not usually visible; and the treatment is effective.

The type of clinical presentations of CL caused by *L. infantum* in Majorca means that on many occasions, it is not suspected, and diagnostic methods to determine the presence of the parasite are not carried out, leading to underdiagnosis. Although CL is more frequent than the visceral form, the number of declared VL cases is higher because of its higher morbidity.

The implementation of an algorithm (Figure S1) in all hospitals in the Balearic Islands would allow the same protocol to be followed in the event of a lesion suspected to be CL. In this way, the true incidence of the disease could be assessed, and the problem of underreporting minimized.

## Figures and Tables

**Table 1:** Number and incidence of cutaneous leishmaniasis cases according to the Bulletin of the Epidemiological Surveillance Network of the Balearic Islands compared to those diagnosed in the Manacor Hospital of Majorca in 2013–2017 [8,9].

	Balearic Islands		Manacor Hospital (County of Llevant)	
Year	CL	Cases/100,000 Inhabitants	CL	Cases/100,000 Inhabitants
2013	12	1.4	39	26
2014	17	2.2	17	11.3
2015	28	3.3	26	17.3
2016	30	3.3	24	16
2017	22	3.8	20	13.3
**Total**	**109**		**126**	

**Table 2:** Suspected clinical diagnosis of cases by age groups.

Age Group	Suspected Diagnosis	M	F	Total
<18 years	Boil		1	1
	Mass cell tumor	1		1
	Panniculitis		1	1
	Granulomatous rosacea		1	1
	Spitz’s nevus		1	1
	Xanthogranuloma	1		1
	Leishmaniasis	9	14	23
Total	Total	11	18	29
18–65 years	Basal cell carcinoma		1	1
	Squamous cell carcinoma		1	1
	Scar	1		1
	Eczema	1		1
	Granulomatous disease		1	1
	Erysipelas		1	1
	Boil		1	1
	Boil/tub		1	1
	Pyogenic granuloma		1	1
	Mycoses		1	1
	Lymphoma	1	1	2
	Lichen planus	1		1
	Amelanotic melanoma		1	1
	Mycobacteria		1	1
	Mycobacteria/fungus		1	1
	Bite		1	1
	Pyoderma gangrenosum		1	1
	Sore	1		1
	Wart/nevus		1	1
	Leishmaniasis	27	17	44
Total	Total	32	32	64
>65 years	Basal cell carcinoma	2	1	3
	Squamous cell carcinoma	2		2
	Folliculitis	1		1
	Folliculitis/scar	1		1
	Actinic granuloma		1	1
	Lymphoma		1	1
	Lipoma	1		1
	Mycobacteria		1	1
	Nevus/bite		1	1
	Cyst	1		1
	Sarcoidosis		1	1
	Tub		1	1
	Leishmaniasis	14	4	18
Total	Total	22	11	33
Total general		65	61	126

**Table 3:** Distribution of CL cases by age and sex.

	Total		Age Group	
		<18	18–65	>65
**Number of cases, *n* (%)**	126 (100)	29 (23.0)	64 (50.8)	33 (26.2)
**Sex**				
Woman, *n* (%)	65 (51.6)	11 (8.7)	32 (25.4)	22 (17.5)
Man, *n* (%)	61 (48.4)	18 (14.3)	32 (25.4)	11 (8.7)
**Age**				
Mean ± SD, years	44.63 ± 25.50	6 ± 4	56 ± 4	75 ± 1
Median, years	49	5	55	57
Range, years	0–86	0–16	19–64	65–86

**Table 4:** Type of lesion according to the sex and age of patients (*n* = 126).

Clinical Picture	Lesion Type	Sex		Total	Age		
		F	M		<18	18–65	>65
**Single**	**Papule, *n***	29	24	53	14	22	17
	Plaque, *n*	25	23	49	8	27	14
Total, *n* (%) [CI 95%]		55 (84.6) [73.7–91.6]	47 (77) [64.9–85.9]	102 (81) [73.2–86.9]	22 (75.9) [57.6–88]	49 (76.6) [64.8–85.4]	31 (93.9) [79.4–99.3]
Multiple *	Papule, *n*	7	4	11	2	8	1
	Plaque, *n*	2	9	11	5	5	1
Total, *n* (%) [CI 95%]		9 (13.8) [7.2–24.5]	13 (21.3) [12.8–33.3]	22 (17.5) [11.8–25.1]	7 (24.1) [12–42.4]	13 (20.3) [12.1–31.9]	2 (6.1) [0.7–20.6]
Mucosa	Sore, *n*	1	1	2	0	2	0
Total, *n* (%) [CI 95%]		1 (1.5) [0.01–9]	1 (1.6) [0.01–9.6]	2 (1.6) [0.1–0.6]	0	2 (3.1) [0.2–11.3]	0
Overall total		65	61	126	29	64	33

* The data refer to the first lesion in case of multiple lesions. F: female; M: male.

**Table 5:** Location and type of lesions according to the sex and age of the patients (*n* = 126).

Location	Lesion Type	Sex		Total	Age		
		F	M		<18	18–65	>65
HN	Papule	22	16	38	14	12	12
	Plaque	17	14	31	11	9	11
	Sore	1	1	2	0	2	0
Total HN		40	31	71	25	23	23
UE	Papule	7	4	11	0	10	1
	Plaque	7	11	18	0	15	3
Total UE		14	15	29	0	25	4
LE	Papule	3	8	11	2	4	5
	Plaque	3	4	7	1	5	1
Total LE		6	12	18	3	9	6
T	Papule	4	0	4	0	4	0
	Plaque	1	3	4	1	3	0
Total T		5	3	8	1	7	0
Total HN + UE + LE + T		65	61	126	29	64	33

HN, head and neck; LE, lower extremities; UE, upper extremities; T, trunk.

**Table 6:** Lesion size * by sex and age (*n* = 126).

Size, mm	Sex		Total, *n* (%)	Age		
	F	M		<18	18–65	>65
1–5	12	9	21 (16.6)	8	11	2
6–10	40	29	69 (54.7)	12	39	18
11–15	9	16	25 (19.9)	8	8	9
16–20	3	3	6 (4.8)	0	5	2
>20	1	4	5 (4)	1	2	2
Total, *n*	65	61	126	29	64	33

* In case of multiple lesions, the size of the first lesion is included.

## Data Availability

The data presented in this study are available on request from the corresponding author.

## Supplementary Materials

The following supporting information can be downloaded at: https://www.mdpi.com/article/10.3390/microorganisms11010126/s1, Figure S1: Diagnosis and notification algorithm for Cutaneous Leishmaniasis.
