# Supplementary material for: Underestimation of Human Cutaneous Leishmaniasis Caused by Leishmania infantum in an Endemic Area of the Mediterranean Basin (Balearic Islands)
Source: Microorganisms. 2023 Jan 4;11(1):126. doi: 10.3390/microorganisms11010126 (PMC9867513; doi:10.3390/microorganisms11010126)
Supplement: Supplementary file 1 [file microorganisms-11-00126-s001.zip › microorganisms-2102451-supplementary.pptx]

## Slide 1
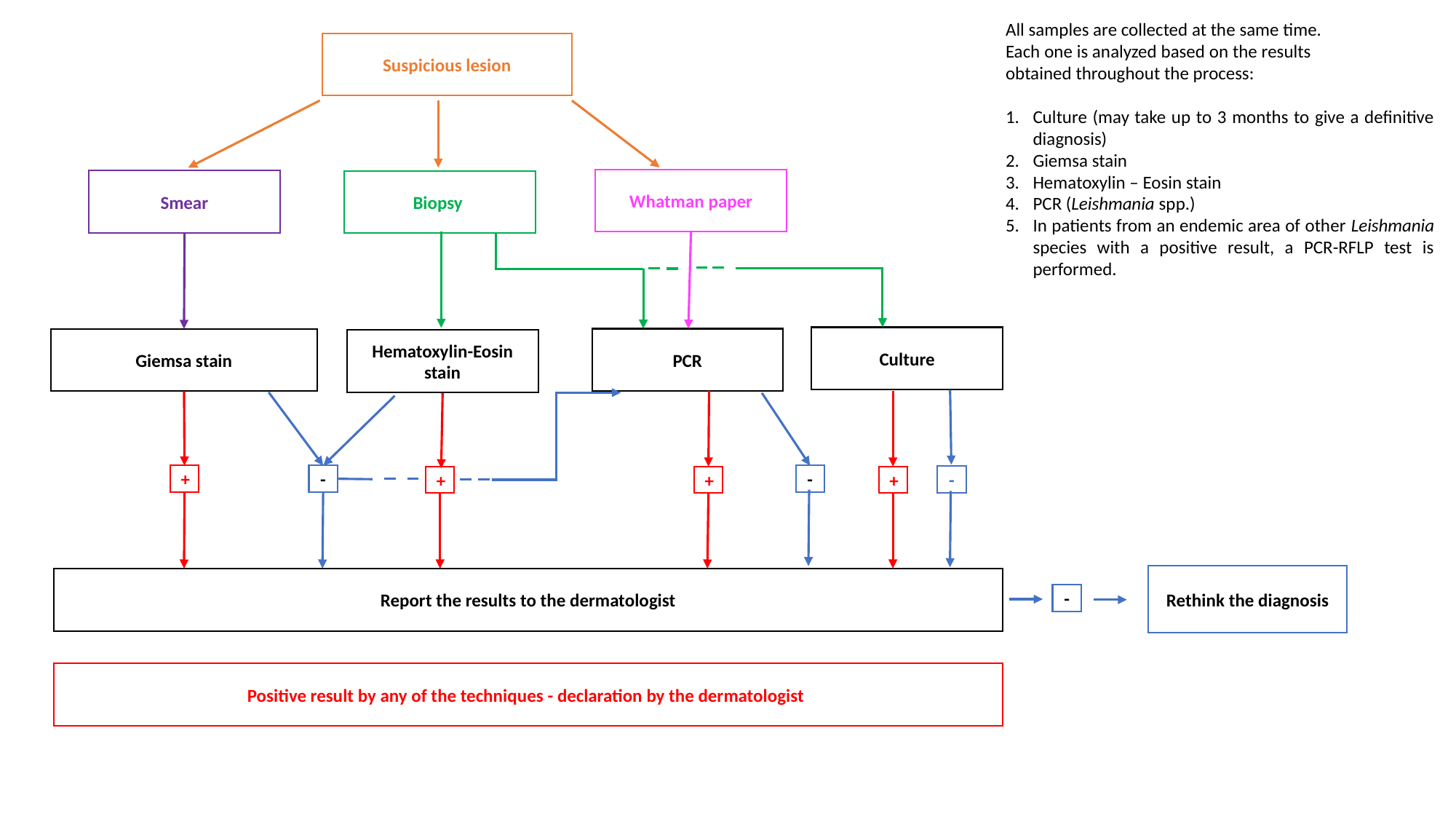

All samples are collected at the same time.
Each one is analyzed based on the results
obtained throughout the process:
Culture (may take up to 3 months to give a definitive diagnosis)
Giemsa stain
Hematoxylin – Eosin stain
PCR (Leishmania spp.)
In patients from an endemic area of other Leishmania species with a positive result, a PCR-RFLP test is performed.
Suspicious lesion
Whatman paper
Smear
Biopsy
Culture
PCR
Giemsa stain
Hematoxylin-Eosin stain
-
-
+
-
+
+
+
Rethink the diagnosis
Report the results to the dermatologist
-
Positive result by any of the techniques - declaration by the dermatologist
